# Reassessment of oxidative stress in idiopathic sudden hearing loss and preliminary exploration of the effect of physiological concentration of melatonin on prognosis

**DOI:** 10.3389/fneur.2023.1249312

**Published:** 2023-09-06

**Authors:** Jianxiong Song, Fang Ouyang, Yuanping Xiong, Qing Luo, Hongqun Jiang, Li Fan, Zhiyuan Zhang

**Affiliations:** ^1^Department of Otolaryngology, The First Affiliated Hospital of Nanchang University, Nanchang, China; ^2^Department of Endocrinology, The First Affiliated Hospital of Nanchang University, Nanchang, China

**Keywords:** idiopathic sudden sensorineural hearing loss, reactive oxygen species, melatonin, total antioxidant capacity, oxidative stress

## Abstract

**Background and purpose:**

The pathogenesis of idiopathic sudden sensorineural hearing loss (ISSNHL) is still unclear, and there is no targeted treatment. This research aimed to verify the role of oxidative stress in ISSNHL and explore whether melatonin has a protective effect on hearing.

**Materials and methods:**

A total of 43 patients with ISSNHL and 15 healthy controls were recruited to detect the level of melatonin, reactive oxygen species (ROS), and total antioxidant capacity (TAC) in the blood and compared before and after treatment. Multivariate logistic regression models were performed to assess the factors relevant to the occurrence and improvement of ISSNHL.

**Results:**

The patients with ISSNHL showed significantly higher ROS levels than controls (4.42 ± 4.40 vs. 2.30 ± 0.59; *p* = 0.031). The levels of basal melatonin were higher (1400.83 ± 784.89 vs. 1095.97 ± 689.08; *p* = 0.046) and ROS levels were lower (3.05 ± 1.81 vs. 5.62 ± 5.56; *p* = 0.042) in the effective group as compared with the ineffective group. Logistic regression analysis showed that melatonin (OR = 0.999, 95% CI 0.997–1.000, *p* = 0.049), ROS (OR = 1.154, 95% CI 1.025–2.236, *p* = 0.037), and vertigo (OR = 3.011, 95% CI 1.339–26.983, *p* = 0.019) were independent factors associated with hearing improvement. Besides, the level of melatonin (OR = 0.999, 95% CI 0.998–1.000, *p* = 0.023) and ROS (OR = 3.248, 95% CI 1.109–9.516, *p* = 0.032) were associated with the occurrence of ISSNHL.

**Conclusion:**

Our findings may suggest oxidative stress involvement in ISSNHL etiopathogenesis. The level of melatonin and ROS, and vertigo appear to be predictive of the effectiveness of hearing improvement following ISSNHL treatment.

## 1. Introduction

Idiopathic sudden sensorineural hearing loss (ISSNHL) is a common otological emergency, which refers to a hearing loss of more than 30 dB in three consecutive frequencies within 72 h (1). This disease is frequently diagnosed among people over 40 years old and is accompanied by tinnitus and vertigo (2). It is possible to develop into permanent deafness without timely treatment (3), which will seriously affect the life of patients.

The etiology of ISSNHL is still in the exploratory stage. At present, many scholars believed that it is mainly related to viral infection, endolymphatic hydrops, cochlear microcirculation, immune damage, and psychological factors (4). No matter what the pathogenic factors are, it is worth affirming that the cochlea is sensitive to ischemia and hypoxia (5). This change in the microenvironment aggravates the production of reactive oxygen species (ROS). Related studies suggest that oxidative stress is a risk factor for microcirculation damage (6). ROS are constantly produced during metabolism (7) and transformed into each other in four forms (8). The physiological concentration of ROS is beneficial to the body. They kill pathogenic microorganisms, maintain normal immune function (9), and act as a key second messenger in a variety of cellular signaling pathways (10). Once the balance of the oxidation-antioxidation system is broken, there are adverse effects. ROS directly attack proteins, lipids, and DNA. Furthermore, they change the phenotype of vascular smooth muscle cells, reduce the fluidity of cell membranes (11), and affect cochlear perfusion. There is evidence that endothelial dysfunction and impaired cochlea perfusion play a crucial role in the pathogenesis of ISSNHL (12).

Therefore, neutralizing excessive ROS may be an effective treatment for ISSNHL. At present, melatonin is one of the most investigated antioxidants. It is mainly produced by the pineal gland at night and participates in many physiological metabolic processes, such as regulating hormone secretion, controlling human growth, and aging (13). Compared with other antioxidants, melatonin can easily cross cell barriers due to its oleophilic and hydrophilic structure. Takumida et al. (14) found that melatonin and its receptors (melatonin receptor 1A; MT-1 and melatonin receptor 1B; MT-2) are present in the inner ear, which further supports the hypothesis that melatonin plays a physiological role in the inner ear. Recent studies have confirmed that melatonin has a protective effect on drug-induced hearing loss (DIHL) (15), noise-induced hearing loss (NIHL) (16), and age-related hearing loss (ARHL) (17). Thus, this research aimed to explore whether melatonin has a protective effect on hearing and verify the role of oxidative stress in ISSNHL. Multivariate logistic regression models were performed to assess the factors relevant to the occurrence and improvement of ISSNHL.

## 2. Materials and methods

### 2.1. Participants

This was a prospective study that intended to recruit ISSNHL patients and measure blood parameters. The diagnosis of ISSNHL patients was based on the Clinical Practice Guideline on Sudden Hearing Loss (2012) (18). Patients with the following conditions or diseases were excluded: (1) bilateral ISSNHL; (2) received treatment before admission; (3) the time of onset is more than 10 days; (4) smoking history (>10 cigarettes/day); (5) upper respiratory tract infection within 4 weeks; (6) fluctuating hearing loss; (7) MRI shows acoustic neuroma or other otological diseases; (8) history of ear surgery and ISSNHL; (9) recent history of use of ototoxic drugs; and (10) malignant tumor, hematological disease, diabetes, hypertension, etc. From July to December 2020, a total of 58 ISSNHL patients were treated at the First Affiliated Hospital of Nanchang University. Among them, three patients with diabetes, one patient with hypertension, two patients with bilateral ISSNHL, one patient with contralateral tympanic membrane perforation, four patients had been treated before admission, and four patients refused to participate in this study. Thus, the final analysis included 43 patients. Furthermore, 15 age- and sex-matched healthy participants without a history of otological disease, sudden deafness, and systemic disease were enrolled as controls. Their otology and audiology examinations were normal. The project was approved by the Ethics Committee of the First Affiliated Hospital of Nanchang University [(2019) medical research review no. (100)], and all the participants signed informed consent forms.

### 2.2. Pure tone audiometry and treatment

The patients with ISSNHL were required to sit in a soundproof booth, and air conduction thresholds (from 125 to 8,000 Hz) and bone conduction thresholds (from 250 to 4,000 Hz) were measured. If no reaction was elicited, the maximum sound intensity produced by the audiometer was increased by 5 dB (19). According to the deafness grading standard (20), the average air conduction threshold of 500–4,000 kHz was calculated as the pure-tone average (PTA).

All patients received systemic steroids, vasodilators, and hyperbaric oxygen therapy (HPOT). We rechecked the pure-tone audiometry after treatment. According to hearing recovery, patients were divided into the effective group (more than 15 dB of gain) and the ineffective group (less than 15 dB of gain).

### 2.3. Blood collection

**Figure 1:**
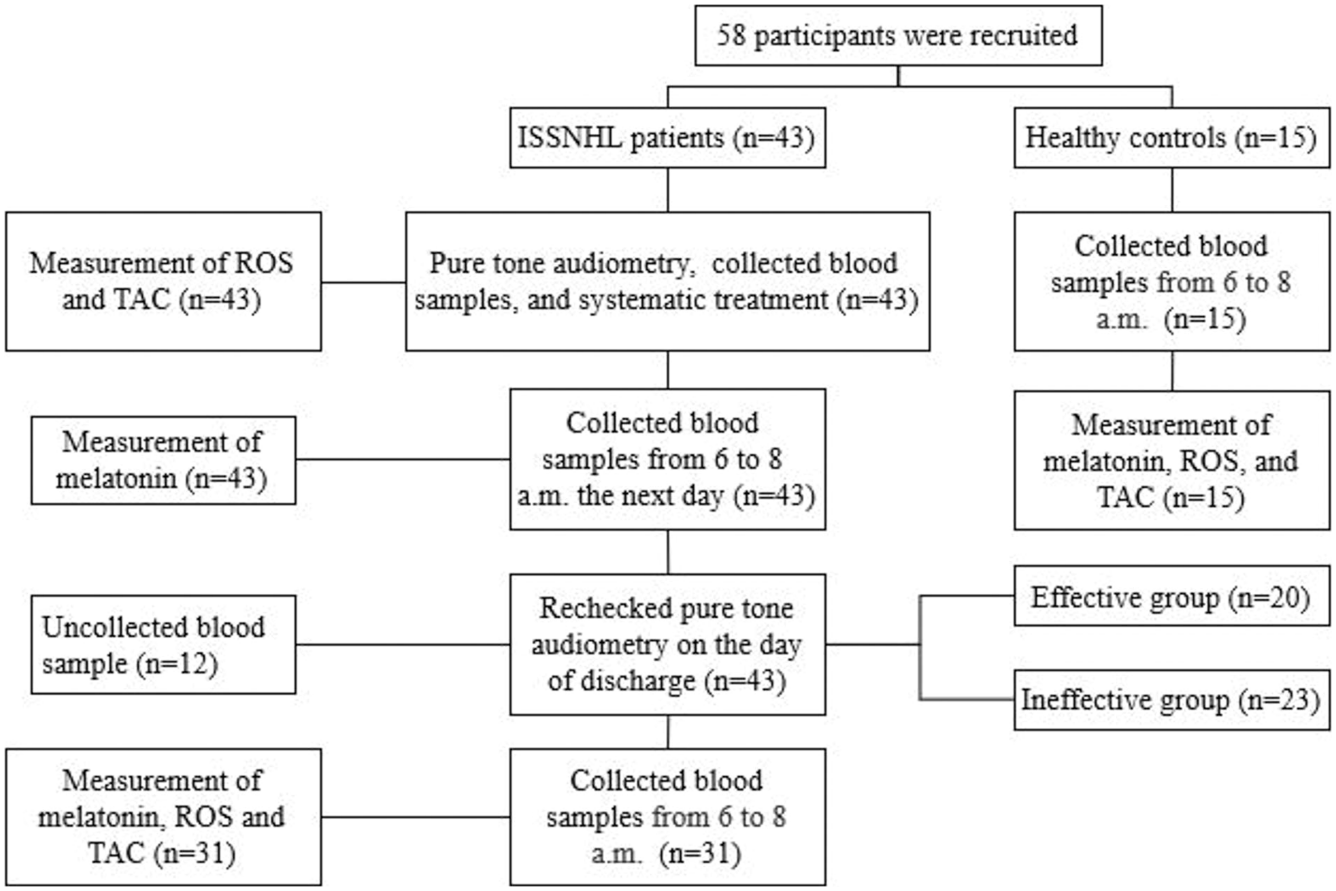
Experimental flow chart.

Blood samples from the patients were collected at the time of admission, from 6 to 8 a.m. on the second day of admission [the secretion of melatonin is relatively stable from 6 to 8 a.m. (21)], and from 6 to 8 a.m. on the day of discharge. Blood samples from the healthy controls were collected from 6 to 8 a.m. The specific operation steps are shown in Figure 1. All blood samples were centrifuged for 10 min at 4°C at a speed of 2,500 rpm within 1 h. The serum was separated and immediately stored in the refrigerator at −80°C. All operations were carried out in the absence of light as far as possible.

### 2.4. Measurement of parameters

#### 2.4.1. Measurement of melatonin

The melatonin levels of the samples were measured by the competitive-ELISA principle using a human melatonin ELISA kit (Nanjing Jiancheng Biological Engineering Research Institute, China). Avidin conjugated to Horseradish Peroxidase (HRP) was added to each microplate well and incubated. Then the 3,3′,5,5′-Tetramethylbenzidine (TMB) substrate solution was added to each well. The enzyme-substrate reaction was terminated by the addition of a stop solution and the color change was measured spectrophotometrically at a wavelength of 450 nm.

#### 2.4.2. Measurement of ROS

ROS levels were measured by using a human ROS ELISA kit (Xiamen Huijia Biotechnology Co., Ltd., China). There are several types of ROS, namely superoxide anion radical, singlet oxygen, hydrogen peroxide, and hydroxyl radical (8). In this study, the human total ROS levels were measured. This ELISA kit uses the double antibody sandwich method. The sample was added to the purified human ROS antibody and combined with the HRP-labeled ROS antibody to form the antibody–antigen–enzyme labeled antibody complex. TMB was then added to form a colored complex, and the absorbance was determined at 450 nm.

#### 2.4.3. Measurement of total antioxidant capacity

Total antioxidant capacity (TAC) was measured by the spectrophotometric method using a commercial kit (Nanjing Jiancheng Biological Engineering Research Institute, China). 2,2′-Azinobis-(3-ethylbenzthiazoline-6-sulphonate) (ABTS) produces green ABTS^+^ under the action of appropriate oxidants. In the presence of antioxidants, the production of ABTS^+^ is inhibited. The TAC of the samples was determined and calculated by measuring the absorbance of ABTS^+^ at 405 or 734 nm.

### 2.5. Statistical analysis

The continuous data were shown as mean ± standard deviation and categorical variables were expressed as frequencies and percentages. Student’s *t*-test was used for the continuous variables with normal distribution, and those with non-normal distribution were compared with the Mann–Whitney *U*-test or Wilcoxon test when applicable. The Chi-square test was used to compare categorical data. Through the above methods, the factors that may affect the occurrence and prognosis of ISSNHL were obtained. Before the regression analysis, we performed a logarithmic conversion of the variables. The BOX-Tidwell method was used to detect whether there was a linear relationship between the logi-conversion values of the independent variables and the dependent variables. In addition, we also needed to verify whether there was multicollinearity between the independent variables. Melatonin, ROS, TAC, and vertigo were taken as the independent variables, and the occurrence and prognosis of ISSNHL were taken as the dependent variables. Multivariate logistic regression models were performed to assess the factors relevant to the occurrence, severity, and improvement of ISSNHL. The odds ratio (OR) and its 95% confidence interval (CI) were estimated for these factors. A *value of p* less than 0.05 was considered to be statistically significant. The SPSS 25.0 software package was used for the analyses.

## 3. Results

**Figure 2:**
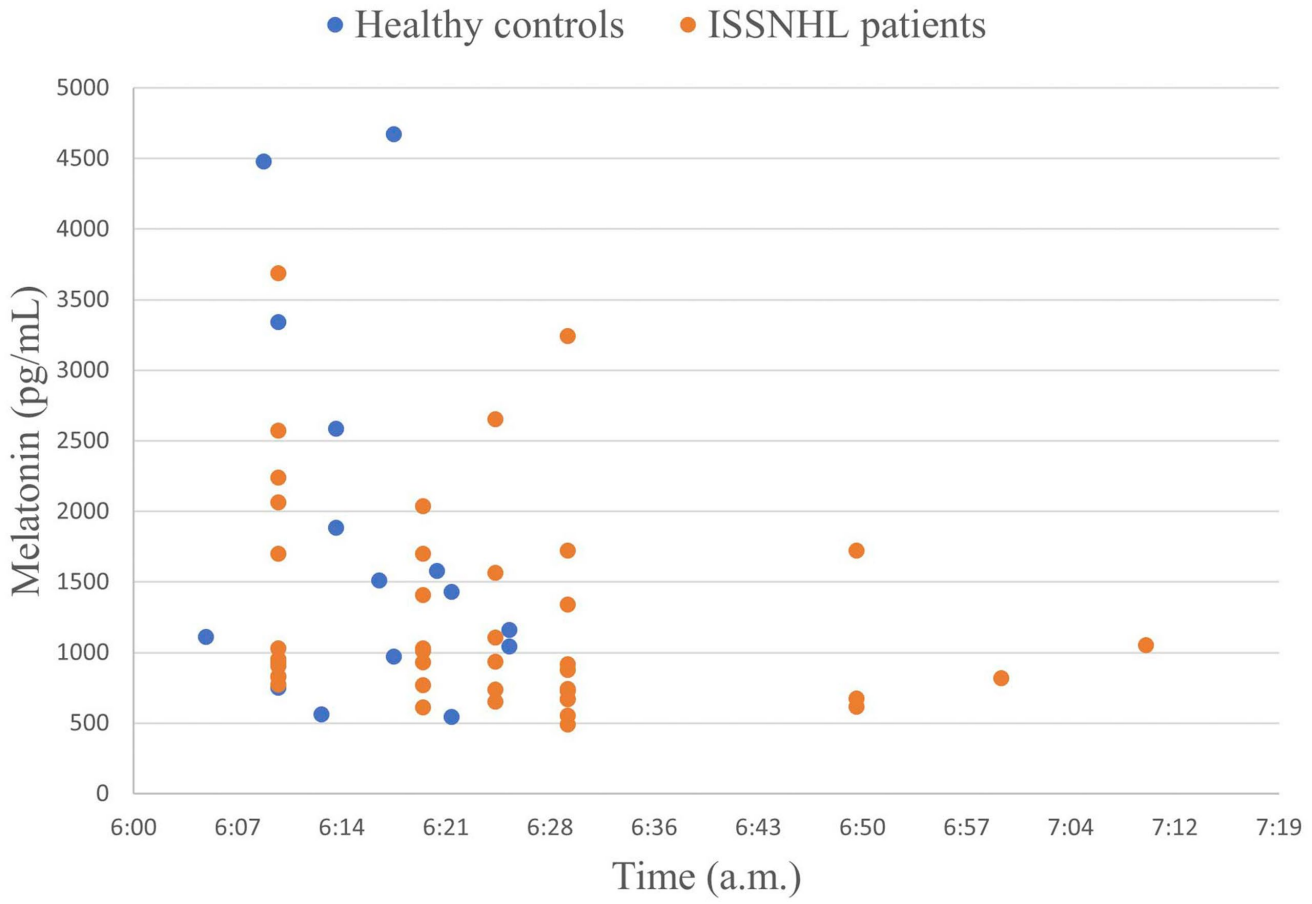
The time of drawing blood and melatonin concentration of each participant.

The time of drawing blood and the corresponding concentration of melatonin for each participant are shown in Figure 2. In addition, the intra-group comparison between the healthy controls group and the ISSNHL patients group showed that the *p*-values were 0.502 and 0.182, respectively, which was not statistically significant. The results show that melatonin secretion was stable during the period in which we drew blood. On admission, the comparison of related factors between the ISSNHL and the control groups is shown in Table 1. The mean ROS levels were significantly higher in the patients than in the controls (4.42 ± 4.40 vs. 2.30 ± 0.59 ng/mL; *p* = 0.031). There was no statistical difference in melatonin and TAC between the two groups, but the basic level of melatonin in the healthy control group was higher than that in the patient group (1841.87 ± 1336.57 vs. 1237.77 ± 742.35 pg/mL).

**Table 1:** Characteristics of the patients with ISSNHL and healthy controls.

	ISSNHL Group (*n* = 43)	Control Group (*n* = 15)	*p-*value
Age (years)	35.63 ± 16.62	34.67 ± 15.50	0.845
Gender (male)	32 (74.42%)	8 (53.3%)	0.129
Melatonin (pg/mL)	1237.77 ± 742.35	1841.87 ± 1336.57	0.077
ROS (ng/mL)	4.42 ± 4.40	2.30 ± 0.59	0.031^*^
TAC (Mm)	0.67 ± 0.08	0.66 ± 0.08	0.617

ROS, reactive oxygen species; TAC, total antioxidant capacity; ^*^indicates that the difference is statistically significant.

According to the significance level (Table 2), there was a linear relationship between the logi-conversion values of all the continuous independent variables and dependent variables. And there was no multicollinearity between each independent variable (Tolerance > 0.1, VIF < 10, Table 3). Subsequently, we used multivariable logistic regression to evaluate the effects of melatonin, ROS, and TAC levels on ISSNHL. Finally, the Logistic model was statistically significant (*X*^2^ = 16.311 *p* < 0.05). The model could correctly classify 79.3% of the research objects. The sensitivity, specificity, positive predictive value, and negative predictive value of the model were 95.3%, 33.3%, 80.4%, and 71.4%, respectively. Among the three independent variables included in the model (Table 4), melatonin (OR = 0.999, 95% CI 0.998–1.000, *p* = 0.023) and ROS (OR = 3.248, 95% CI 1.109–9.516, *p* = 0.032) were statistically significant. The lower the level of melatonin, the higher the level of ROS, and the higher the risk of ISSNHL.

**Table 2:** Test of the linear relationship of the variables.

	*p*-value
Melatonin by ln_melatonin	0.845
ROS by ln_ROS	0.129
TAC by ln_TAC	0.077

ROS, reactive oxygen species; TAC, total antioxidant capacity; Ln_, logi-conversion values of independent variables.

**Table 3:** Multiple collinearity test of the variables.

	Tolerance	VIF
Melatonin	0.910	1.099
ROS	0.952	1.050
TAC	0.875	1.143

ROS, reactive oxygen species; TAC, total antioxidant capacity; VIF, variance expansion factor.

**Table 4:** Association between the relevant factors and ISSNHL occurrence: logistic regression analysis.

	B	SE	Wald	*P*	OR	Lower	Upper
Melatonin	−0.01	<0.01	5.16	0.023^*^	0.999	0.998	1.000
ROS	1.18	0.55	4.62	0.032^*^	3.248	1.109	9.516
TAC	3.20	5.01	0.41	0.523	24.503	0.001	453674.325

ROS, reactive oxygen species; TAC, total antioxidant capacity; ^*^indicates that the difference is statistically significant.

The general status of the patients in the effective and ineffective groups is shown in Table 5. The effective group had lower basic ROS levels (3.05 ± 1.81 vs. 5.62 ± 5.56 ng/mL; *p* = 0.042) and higher melatonin levels (1400.83 ± 784.89 vs.1095.97 ± 689.08 pg/mL; *p* = 0.046). Furthermore, there were statistical differences in vertigo (*X*^2^ = 6.702, *p* = 0.010) between these two groups.

**Table 5:** Characteristics of the effective and ineffective groups.

	Effective group (*n* = 20)	Ineffective group (*n* = 23)	*p*-value
Age (years)	32.75 ± 17.41	38.13 ± 15.86	0.295
Gender (male)	32 (74.42%)	8 (53.3%)	0.129
Side (left)	8 (40%)	13 (56.52%)	0.280
Days of onset	3.00 ± 1.75	4.22 ± 2.83	0.103
Tinnitus	20 (100%)	23 (100%)	-
Vertigo	6 (30%)	16 (69.57%)	0.010^*^
PTA (dB)	68.70 ± 24.67	78.22 ± 21.64	0.185
Melatonin (pg/mL)	1400.83 ± 784.89	1095.97 ± 689.08	0.046^*^
ROS (ng/mL)	3.05 ± 1.81	5.62 ± 5.56	0.042^*^
TAC (Mm)	0.68 ± 0.08	0.66 ± 0.08	0.606

PTA, pure tone average; ROS, reactive oxygen species; TAC, total antioxidant capacity; ^*^indicates that the difference is statistically significant.

Again, we performed the same verification as the steps above. Multivariable logistic regression was used to analyze the relationship between melatonin, ROS, vertigo, and the prognosis of ISSNHL. Finally, the Logistic model was statistically significant (*X*^2^ = 16.499, *p* < 0.05). The model could correctly classify 76.7% of the research objects. The sensitivity, specificity, positive predictive value, and negative predictive value of the model were 78.3%, 75.0%, 75.0%, and 78.3%, respectively. Among the three independent variables included in the model (Table 6), melatonin (OR = 0.999, 95% CI 0.997–1.000, *p* = 0.049), ROS (OR = 1.154, 95% CI 1.025–2.236, *p* = 0.037), and vertigo (OR = 6.011, 95% CI 1.339–26.983, *p* = 0.019) were statistically significant. The lower the level of melatonin, the higher the level of ROS, accompanied by vertigo, and the higher the risk of poor prognosis for the patients with ISSHNL.

**Table 6:** Association between the relevant factors and hearing improvement: logistic regression analysis.

	B	SE	Wald	*P*	OR	Lower	Upper
Melatonin	−0.01	<0.01	3.87	0.049^*^	0.999	0.997	1.000
ROS	0.42	0.20	4.35	0.037^*^	1.514	1.025	2.236
Vertigo	1.79	0.77	5.48	0.019^*^	6.011	1.339	26.983

ROS, reactive oxygen species; ^*^indicates that the difference is statistically significant.

**Figure 3:**
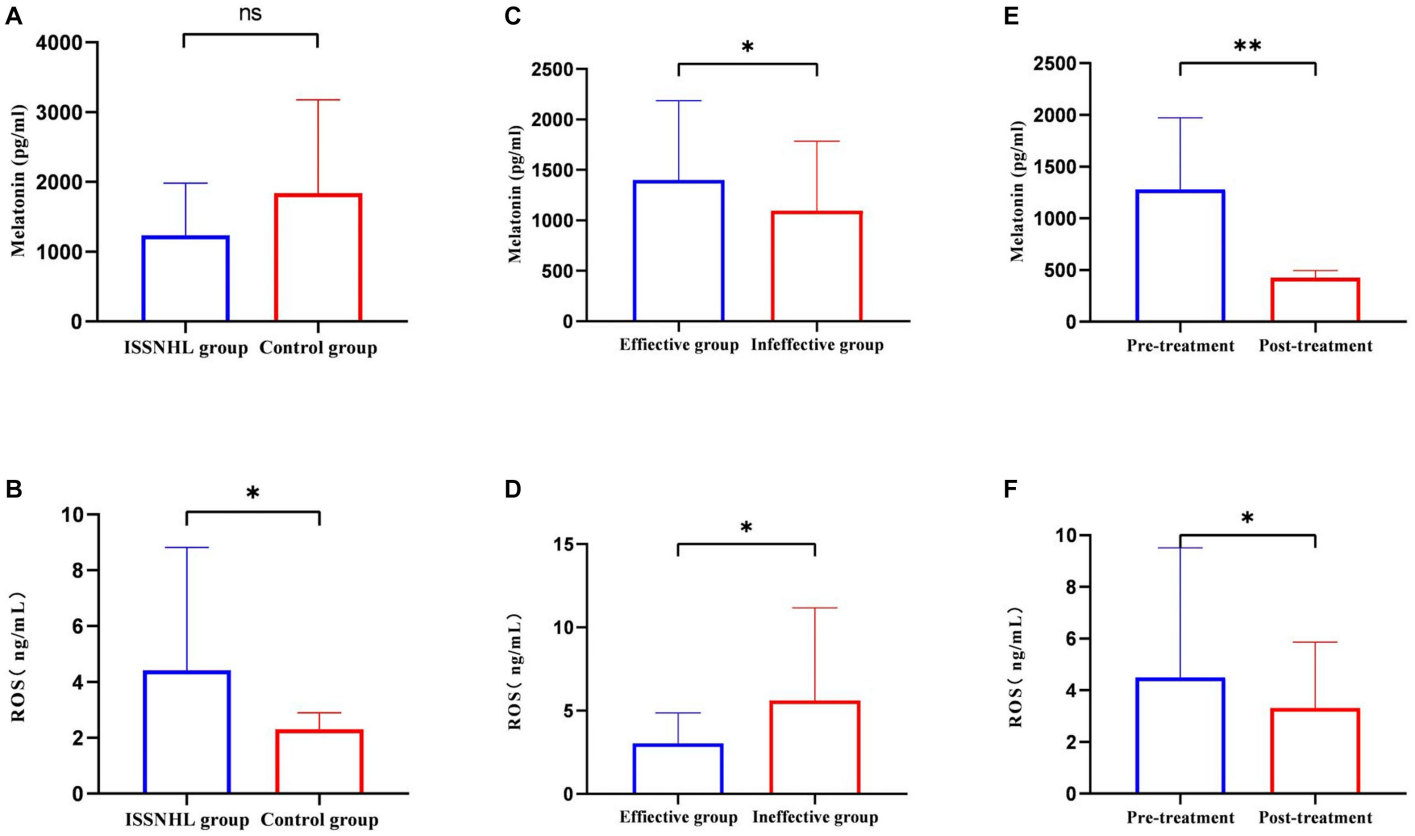
The intuitive comparison of melatonin and ROS in each group. **(A)** Comparison of melatonin levels between the ISSNHL group and the control group. **(B)** Comparison of ROS levels between the ISSNHL group and the control group. **(C)** Comparison of melatonin levels before treatment between the effective group and the ineffective group. The average blood sampling time of the effective group and the ineffective group was 6:21 ± 14 and 6:28 ± 13 in the morning. **(D)** Comparison of ROS levels before treatment between the effective group and the ineffective group. **(E)** Comparison of melatonin levels in ISSNHL patients before and after treatment. The average blood sampling time before and after treatment was 6:22 ± 13 and 6:27 ± 13 in the morning. **(F)** Comparison of ROS levels in ISSNHL patients before and after treatment. */**indicates that the difference is statistically significant.

After systemic treatment, blood samples were collected again in 31 patients. Comparing the three indicators before and after treatment (Table 7), the melatonin levels were significantly reduced (1279.83 ± 691.29 vs. 425.24 ± 69.14 pg/mL; *p* < 0.01), the TAC was enhanced (0.69 ± 0.08 vs. 0.83 ± 0.09 Mm; *p* < 0.01), and the body’s state of high oxidative stress had been alleviated (4.50 ± 5.02 vs. 3.32 ± 2.54 ng/mL; *p* = 0.034). Compared with the control group (Table 8), the level of ROS in the patients with ISSNHL decreased to normal after treatment. For more information on the intuitive comparison of various indicators, please refer to Figure 3.

**Table 7:** Comparison of various indicators before and after treatment.

	Pre-treatment group (*n* = 31)	Post-treatment group (*n* = 31)	*p*-value
Melatonin (pg/mL)	1279.83 ± 691.29	425.24 ± 69.14	<0.01^**^
ROS (ng/mL)	4.50 ± 5.02	3.32 ± 2.54	0.034^*^
TAC (Mm)	0.69 ± 0.08	0.83 ± 0.09	<0.01^**^

ROS, reactive oxygen species; TAC, total antioxidant capacity; */**indicates that the difference is statistically significant.

**Table 8:** Comparison of various indicators between patients with ISSNHL after treatment and healthy controls.

	Control group (*n* = 15)	Post-treatment group (*n* = 31)	*p*-value
Melatonin (pg/mL)	1841.87 ± 1336.57	425.24 ± 69.14	<0.01^**^
ROS (ng/mL)	2.30 ± 0.59	3.32 ± 2.54	0.815
TAC (Mm)	0.66 ± 0.08	0.83 ± 0.09	<0.01^**^

ROS, reactive oxygen species; TAC, total antioxidant capacity; ^**^indicates that the difference is statistically significant.

## 4. Discussion

The influence of ISSNHL on the personal life of patients promotes the continuous development of research on the pathogenesis of ISSNHL. This study suggests that the level of melatonin and ROS may be related to the occurrence of ISSNHL. The logistic regression analysis indicated that melatonin, ROS, and vertigo were significantly correlated to hearing improvement after ISSNHL treatment. Therefore, improving the state of oxidative stress may become an effective method for the treatment of ISSNHL in the future.

The results show that the ROS levels of patients with ISSNHL were higher than that of the healthy controls (Figure 3B), which is consistent with the results in the literature (22, 23). This is not a coincidence as Guo et al. (24) speculated that the generation of superoxide anion radicals and the decreased activity of nitric oxide synthase led to endothelial dysfunction. Finally, hair cell loss, vascular intimal thickening, and luminal narrowing of spiral arteries were detected in the mouse cochlea. Merchant et al. (25) pointed out that the pathological activation of nuclear factor NF-kappa-B (NF-kB) induces oxidative stress, which causes ISSNHL. In a prospective study, total oxidative state (TOS) levels in 50 patients with ISSNHL were compared with those in 50 normal controls, and the corresponding global oxidative stress index was calculated. The patients with ISSNHL showed significantly higher TOS levels and oxidation index (26). Excessive ROS depletes intracellular nitric oxide levels and increases the release of adhesion molecules, lipid-inflammatory mediators, and cytokines, leading to endothelial damage (6) and blood flow disorders. The cochlear labyrinthine artery is sensitive to ischemia as it has no branches. Subsequently, impairment of perfusion leads to an immediate dysfunction of the organ of Corti, resulting in irreversible hearing loss. ROS also have toxic effects on cell functions, especially hydroxyl radicals, which directly damage various organelles and pathways. As the main source of ROS, mitochondria may be damaged while producing ROS, which affects mitochondrial membrane potential and energy metabolism (27). Moreover, Becatti et al. (28) used fluorescent markers to evaluate the fluidity of red blood cell membranes. The results showed that lipid peroxidation of red blood cell membranes in patients with ISSNHL resulted in decreased deformability and increased blood viscosity, affecting the cochlear microcirculation.

According to other authors, oxidative stress is thought to play a role in sensorineural hearing loss (SHL) (29). ROS have been identified as the main cause of aminoglycoside antibiotics-induced hearing loss (30). Besides, ROS induce the reduction of nicotinamide adenine dinucleotide phosphate (NADPH) by binding to the sulfhydryl group of the enzyme and affecting glutathione peroxidase (GPX) and glutathione reductase (GRD) activity (31), resulting in the conversion of hydrogen peroxide to hydroxyl radicals instead of water. Furthermore, the antioxidant defense mechanism of hair cells is weakened, which leads to the release of cytochrome c from mitochondria and activates the caspase pathway to trigger cell apoptosis (32) and causes bilateral high-frequency SHL. A guinea pig model (33) showed that ROS transduced the mitogen-activated protein kinase (MAPK) pathway to induce hair cell apoptosis after sound injury. It is uncertain whether these verified pathways apply to ISSNHL and the molecular mechanism of the role of ROS in ISSNHL is still being explored.

We found that the ROS levels in the ineffective group were higher than those in the effective group (Figure 3D), and regression analysis showed that the higher the ROS, the worse the prognosis of ISSNHL. Higher concentrations of ROS may cause more hair cell damage and at a certain peak level, endothelial cells undergo “sudden death” (34). For this reason, the hearing loss that has already occurred is hard to recover, even when prompt and comprehensive treatment is administered. Furthermore, the basal region of the cochlea responsible for high-frequency hearing is more metabolically active than the apical region. Therefore, the basal region generates more ROS while consuming energy, and is more susceptible to oxidative stress damage (35). This may also be the reason for the prognosis of the all-frequency and high-frequency hearing loss being worse than other types of ISSNHL.

There was no significant difference in TAC between the patients with ISSNHL and the healthy controls (Table 8). We believe that the normal antioxidant system could not remove the excess ROS, and the balance had been broken. After systemic treatment, the level of ROS dropped to the same level as the healthy controls (Table 8) and the antioxidant capacity was enhanced. Consistent with the results of previous findings (36), the state of oxidative stress *in vivo* was significantly improved after steroid treatment. Glucocorticoids (GCs) effectively improve the microcirculation of the inner ear, decrease the edema of vascular endothelial cells, and improve the state of ischemia and hypoxia (37). After treatment, the level of ROS decreased (Figure 3F), which further shows that cochlear microcirculation is related to ROS. Unfortunately, only a part of the patients had improved hearing. We postulate the following reasons: first, ROS are not the only causative agent, and many other factors are involved in the pathogenesis of ISSNHL; second, hair cells could not regenerate after being destroyed by ROS; third, it takes a longer period for the hearing improvement of ISSNHL patients.

It is certain that oxidative stress causes damage by inducing endothelial dysfunction within inner ear micro-circulation. To eliminate excessive ROS to improve the hearing of patients, we chose to observe serum melatonin concentrations as it, as an antioxidant, has been proven to protect hair cells in SHL. Demir et al. (15) injected melatonin into the tympanic chamber of rats and found that melatonin has an antagonistic effect on cisplatin ototoxicity due to its antioxidant and immunomodulatory functions. Serra et al. (17) obtained the same results in a study on melatonin and ARHL. Mice with orally administered melatonin maintained a higher distortion product otoacoustic emissions (DPOAE) amplitude and signal-to-noise ratio at the end of the experiment. Melatonin is an indoleamine hormone secreted by the pineal gland controlled by the hypothalamic suprachiasmatic nucleus (SCN). Compared with other traditional antioxidants, melatonin has certain advantages. Its structural properties enable it to shuttle cells freely, effectively reducing high levels of ROS.

Our research found that the melatonin levels in the effective group were higher than those in the ineffective group (Figure 3C) before treatment. We speculate that a higher concentration of basal melatonin may have a positive effect on patients with ISSNHL, as shown by the logistic regression analysis. As previously described, the erythrocyte membranes of patients with ISSNHL are accompanied by lipid peroxidation and functional alterations. Melatonin can directly eliminate ROS when in contact with the cell membrane. At the same time, melatonin reduces intracellular malondialdehyde (MDA, excessive MDA alters membrane fluidity) in a concentration-dependent manner (38), which protects the morphology and function of cell membranes from ROS attack and improves the blood and oxygen supply to the inner ear. Melatonin is then metabolized *in vivo* through enzymatic and chemical reactions such as oxidation to several derivatives, including N1-acetyl-N2-formyl-methoxykynuramine (AFMK) and N2-acetyl-5-methoxykynuramine (AMK) (39). These possess even greater antioxidative capacities than melatonin. In other words, melatonin can scavenge multiple free radicals. In addition, AFMK and AMK exhibit anti-inflammatory and immunomodulatory activities (40), inhibit the formation of prostaglandins, tumor necrosis factor-α (TNF-α), and interleukin-8 (IL-8) (8), and protect hair cells from inflammatory mediators.

The balance of the oxidation-antioxidant system is inseparable from the action of antioxidant enzymes, which is the first line of defense against free radicals. Superoxide dismutase (SOD) is a key antioxidant enzyme that converts superoxide anion radicals into hydrogen peroxide (41). Previous studies have shown that the level of SOD in rats increased significantly after injection with melatonin (42), indicating that melatonin enhanced its activity to achieve indirect antioxidation. In the presence of transition metals, hydrogen peroxide generates highly toxic hydroxyl radicals (43), while catalase (CAT) and glutathione peroxidase (GPX) catalyze peroxidation hydrogen to produce water and oxygen (44). The concentration of GPX is higher at night, which is consistent with the circadian rhythm of melatonin. Melatonin maintains the activity of these enzymes in a receptor-dependent manner. Similarly, Baydas et al. (45) observed a significant decrease in GPX activity in multiple tissues after the removal of the pineal gland in rats. Another study found the activity of CAT is weakened under strong oxidative stress conditions, and when melatonin is present, the high activity of CAT can be maintained (46). The antioxidants can accelerate ROS degradation, while antioxidant enzymes reduce ROS production. Therefore, melatonin not only removes the generated ROS but also avoids the generation of ROS at the source, a characteristic that other antioxidants do not possess.

Mitochondria are the major source of cellular ROS (47). ROS are formed continuously by electron leakage from the respiratory chains (48). Over time, mitochondria may be affected by severe oxidative stress. Therefore, mitochondria may be a potential target for melatonin to eliminate ROS. First, melatonin stabilizes the inner membrane and maintains the integrity of the mitochondria. It then upregulates the expression and activity of antioxidant enzymes in the mitochondria (49) and inhibits pro-oxidant enzymes. Most importantly, melatonin reduces the production of free radicals during respiration by stimulating complexes I and IV of the mitochondrial respiratory chain. Finally, melatonin regulates mitochondrial gene expression and maintains a high level of glutathione, which greatly enhances the antioxidant capacity of mitochondria (50). In short, melatonin could reduce ROS generation and protect the mitochondria from ROS-induced damage.

Most treatments for ISSNHL are based on two etiological theories: circulatory disorders and inflammatory response. Interestingly, the systematic treatment we use seems to have the same effect as melatonin (Table 9). Hargunani et al. (51) confirmed that GCs plays a therapeutic role by binding to glucocorticoid receptors (GRs). GRs are widely found in the human inner ear, especially the spiral ligament in the cochlea (52). Studies have shown that melatonin plays a role in delaying cell loss in spiral ligaments (53). In other words, the concentration of melatonin affects the efficacy of GCs in the treatment of ISSNHL. In addition to GCs as a first-line treatment, most doctors in our country use vasodilators as a combination therapy (54). The best arterial blood supply and adequate oxygenation are prerequisites for the full function of the inner ear, which is the reason for choosing vasodilators. However, melatonin seems to have a superior performance in dilating blood vessels. Melatonin regulates the central nervous system and coordinates the activity of multiple organs to maintain more regular and stable hemodynamics. It can also promote the production of nitric oxide (NO) and reduce peripheral resistance and ischemia–reperfusion injury (55, 56). When to use HPOT is still controversial. Our view is consistent with some recent studies (57, 58) that additional therapeutic benefit can be provided when HPOT is used in combination with GCs to treat ISSNHL. It is undeniable that HBOT has the function of increasing the oxygen tension in the blood and nourishing nerve elements, whether as a routine treatment or as a remedial measure (59). Besides, some domestic scholars have pointed out that HPOT can activate the superoxide free radical scavenging system and reduce the damage to the inner ear caused by ROS. However, the use of hyperbaric oxygen (HPO) may also accelerate the production of ROS and lead to lipid peroxidation, which is harmful to the body (60). Surprisingly, it has been reported that melatonin could effectively prevent HPO-induced oxidative stress, even physiologically secreted melatonin (61). Animal experiments have shown that the combination of melatonin and HPO was superior to either one in vascular events (62). Based on these findings and our regression analysis, we boldly speculate that melatonin can cooperate with these treatment measures to play a more active role and avoid some side effects.

**Table 9:** The function of different treatments.

	GC	Vasodilator	HPOT	Melatonin
Regulation of endothelial cell function Relieve vasospasm	√	√		√
Improve microcirculation Improve ischemia and hypoxia	√	√	√	√
Inhibit inflammatory response	√		√	√
Maintain endolymphatic homeostasis Reduce hair cell edema	√		√	
Immunosuppression	√			√
Elimination of ROS Improve the ability of antioxidation			√	√
Nutritive nerve				√

GC, glucocorticoids; HPOT, hyperbaric oxygen therapy; ROS, reactive oxygen species.

After treatment, the concentration of melatonin decreased significantly (Table 7), which may be related to our treatment. The link between steroids and melatonin was mentioned a long time ago. Demisch et al. (63) observed that melatonin plasma levels decreased significantly in adults treated with dexamethasone. They believed that dexamethasone could interact with the pineal gland to affect melatonin secretion. Another study showed that oral GCs can increase melatonin excretion in children (64). With the deepening of the study, the pineal gland was considered a neuroendocrine organ regulated by the central clock (SCN) and peripheral circadian clocks (65). Dexamethasone treatment changed the mRNA expression of several clock genes in the pineal gland. Furthermore, the synthesis of melatonin requires the participation of a variety of enzymes. Arylalkylamine N-acetyltransferase (AANAT) is the rate-limiting enzyme in the synthesis of melatonin (66). Dexamethasone reduces the synthesis of melatonin by reducing the activity of AANAT (67). Other studies have found that propranolol caused a dose-dependent decrease in melatonin levels (68). There are many kinds of vasodilators and their mechanisms are different, so we are not sure whether the vasodilators used to treat ISSNHL will affect the concentration of melatonin. In addition, as mentioned above, melatonin can antagonize the negative effects of HPOT, and endogenous melatonin is even more effective than exogenous administration (69), which may cause melatonin loss. Overall, under the current treatment, first, the synthesis of melatonin was reduced. Second, the excretion of melatonin increased. Third, we postulate that melatonin was involved in the clean-up of ROS and works with drugs. Finally, melatonin decreased significantly after systematic treatment. Of course, there is a lack of research on drug interaction, and the specific mechanism needs to be further studied.

A recent study shows that melatonin alleviates pyroptosis of hair cells via the MT-1 and MT-2/Nrf2 (NFE2L2)/ROS/NLRP3 pathway, which further supports the conjecture that melatonin can be used for the treatment of ISSNHL (70). In addition, melatonin also has an antidepressant function (71) and improves tinnitus (72). It is well known that depression and tinnitus afflict some patients with ISSNHL patients and eliminating these symptoms has a certain positive effect on the prognosis of patients.

Few studies have linked melatonin to ISSNHL. According to our results, appropriate melatonin supplementation may have a positive effect on hearing improvement in patients with ISSNHL.

The limitations of this study include that it was based on the physiological concentration of melatonin and the sample size was too small. Furthermore, the use of peripheral blood measurement indicators to evaluate the redox level of the inner ear was not accurate enough. In future studies, we will establish animal models to measure melatonin and ROS levels in the inner ear with more accurate data.

## 5. Conclusion

We conclude that low plasma melatonin and high ROS are significant in the occurrence and development of ISSNHL. Furthermore, the levels of melatonin and ROS, and vertigo appear to be predictive of the effectiveness of hearing improvement following ISSNHL treatment.

## Data availability statement

The raw data supporting the conclusions of this article will be made available by the authors, without undue reservation.

## Ethics statement

The project was approved by the Ethics Committee of the First Affiliated Hospital of Nanchang University [(2019) medical research review no. (100)]. The studies were conducted in accordance with the local legislation and institutional requirements. Written informed consent for participation in this study was provided by the participants’ legal guardians/next of kin.

## Author contributions

JS and LF designed the experiment, analyzed the data, and wrote the article. FO recruited patients and collected blood samples. YX and QL guided the study. ZZ and HJ gave final approval for the version to be published. All authors contributed to the article and approved the submitted version.

## Funding

This work was supported by the Fund Project of the Education Department of Jiangxi Province (GJJ190028) and the Health Commission Foundation Project of Jiangxi Province (202130198). Central Funds Guiding the Local Science and Technology Development (20221ZDG020066).

## Conflict of interest

The authors declare that the research was conducted in the absence of any commercial or financial relationships that could be construed as a potential conflict of interest.

## Publisher’s note

All claims expressed in this article are solely those of the authors and do not necessarily represent those of their affiliated organizations, or those of the publisher, the editors and the reviewers. Any product that may be evaluated in this article, or claim that may be made by its manufacturer, is not guaranteed or endorsed by the publisher.
